# Laparoscopic rectal cancer resection yields comparable clinical and oncological results with shorter hospital stay compared to open access: a 5-year national cohort

**DOI:** 10.1007/s00384-023-04529-z

**Published:** 2023-10-04

**Authors:** Elisabeth Myrseth, Petter Fosse Gjessing, Linn Såve Nymo, Hartwig Kørner, Jan Terje Kvaløy, Stig Norderval

**Affiliations:** 1 Department of Gastrointestinal Surgery, University Hospital of North Norway, 9019 Tromsø, Norway; 2 Institute of Clinical Medicine, Faculty of Health Science, UiT, The Arctic University of Norway, 9019 Tromsø, Norway; 3 Department of Gastrointestinal Surgery, Stavanger University Hospital, 4068 Stavanger, Norway; 4 Institute of Clinical Medicine, University of Bergen, 5020 Bergen, Norway; 5 Department of Mathematics and Physics, University of Stavanger, 4036 Stavanger, Norway; 6 Department of Research, Stavanger University Hospital, 4068 Stavanger, Norway

**Keywords:** Rectal cancer, Survival, Laparoscopic, Outcome

## Abstract

**Purpose:**

Although widely applied, the results following laparoscopic rectal resection (LRR) compared to open rectal resection (ORR) are still debated. The aim of this study was to assess clinical short- and long-term results as well as oncological resection quality following LRR or ORR for cancer in a 5-year national cohort.

**Methods:**

Data from the Norwegian Registry for Gastrointestinal Surgery and the Norwegian Colorectal Cancer Registry were retrieved from January 2014 to December 2018 for patients who underwent elective resection for rectal cancer. Primary end point was 5-year overall survival. Secondary end points were local recurrence rates within 5 years, oncological resection quality, and short-term outcome measures.

**Results:**

A total of 1796 patients were included, of whom 1284 had undergone LRR and 512 ORR. There was no difference in 5-year survival rates between the groups after adjusting for relevant covariates with Cox regression analyses. Crude 5-year survival was 77.1% following LRR compared to 74.8% following ORR (*p* = 0.015). The 5-year local recurrence rates were 3.1% following LRR and 4.1% following ORR (*p* = 0.249). Length of hospital stay was median 8.0 days (quartiles 7.0–13.0) after ORR compared to 6.0 (quartiles 4.0–8.0) days after LRR. After adjusting for relevant covariates, estimated additional length of stay after ORR was 3.1 days (*p* < 0.001, 95% CI 2.3–3.9). Rates of positive resection margins and number of harvested lymph nodes were similar. There were no other significant differences in short-term outcomes between the groups.

**Conclusion:**

LRR was performed with clinical and oncological outcomes similar to ORR, but with shorter hospital stay.

**Supplementary Information:**

The online version contains supplementary material available at 10.1007/s00384-023-04529-z.

## Introduction

Laparoscopy has eventually become the preferred surgical approach for rectal cancer in many countries [1, 2], although the oncological safety has been a subject for debate. Several studies have shown favorable outcomes after laparoscopic surgery for colon cancer [3–7] with reduced rates of complications and 30-day mortality, and long-term results equal to open access surgery. For rectal cancer, the results are divergent. Some studies have shown favorable or similar short- and long-term results comparing laparoscopic rectal resection (LRR) and open rectal resection (ORR) [8–10], while other studies have reported inferior oncological results following laparoscopy with higher rates of positive circumferential resection margins (CRM) and lower rates of complete excision of mesorectum after TME [11, 12] compared to open access. Only a few studies have explored difference in long-term survival rates and local recurrence rates [13–16].

### NORGAST and the Colorectal Cancer Registry

The long-term results after rectal cancer surgery in Norway are surveyed by the Norwegian Colorectal Cancer Registry. This national quality registry holds data concerning diagnostics, treatment, and follow-up of colorectal cancer patients, and all Norwegian hospitals are obliged to report. The registry has, however, limited information regarding comorbidity and operative and postoperative details. The national quality registry NORGAST (the Norwegian Registry for Gastrointestinal Surgery) was established in 2014, aiming to survey the rate, kind, and severity of complications following major gastrointestinal and hepatobiliary surgery. The registry records selected factors that might affect a surgical outcome such as weight loss, BMI, ECOG status, preexisting severe pulmonary, and cardiac disease as well as operative technique. In addition, short-term postoperative outcome measures including complications, reoperations, length of hospital stay, readmissions, and mortality rates are registered. A detailed presentation of the registry has been published previously [17].

### Registries and data quality

The coverage rate in NORGAST was 75% in 2018, increasing from approximately 20% on a national level in 2014 [18]. Low national coverage rates in the first years of implementation were due to few participating hospitals, but per-hospital coverage among participating hospitals was high. The Norwegian Colorectal Cancer Registry has a coverage rate higher than 90% [19]. Variable completeness varies, with almost 100% completeness in NORGAST compared to 70% for some variables in the Norwegian Colorectal Cancer Registry. The latter registry includes data from various sources, such as clinical reports on diagnosis, treatment, and histopathological reports. However, as both registries overlap on a number of core variables, data linking results in an overall high degree of variable completeness. Patients with missing values were excluded from the specific analysis where data were missing.

Both registries are nationwide with mandatory registration, and have been approved as national quality registers by the National Directory of Health according to defined quality criteria [20]. Both registries validate data against the Norwegian Patient Registry (i.e., the official registry for the national public health service) with yearly control on completeness of data [21, 22]. Some of the data in NORGAST have been validated manually by comparing to electronic medical files for patients included for 3 earlier studies [18]. The Colorectal Cancer Registry has been validated several times, recently in 2022 with near complete data for rectal cancer patients [22].

### Hypothesis and primary and secondary end points

Data from NORGAST combined with data from the Norwegian Colorectal Cancer Registry enabled assessment of both short- and long-term outcomes following rectal cancer surgery adjusting for factors like operative technique, comorbidity, and cancer stage. Several earlier controlled studies have investigated outcomes following laparoscopic and open rectal resections, but this registry-based study aimed to provide information on results from an unselected national cohort, i.e., real-world data, after implementation of minimal invasive treatment for rectal cancer.

The present study hypothesized that results after laparoscopic surgery for rectal cancer would be similar to those after open access surgery, in terms of survival, local recurrence, and short-term outcomes. The aim of this study was to assess the short- and long-term results following elective major rectal resection for rectal cancer based on data from NORGAST and the Norwegian Colorectal Cancer Registry. Primary end point was 5-year overall survival. Secondary end points were local recurrence rates within 5 years, oncological resection quality and short-term outcome measures. The manuscript was drafted in accordance with the STROBE guidelines for observational studies [23].

## Methods

### Study population

Patients who underwent elective major resection for rectal cancer from January 1, 2014, to December 31, 2018, were identified in the NORGAST registry based on the combination of a NSCP (NOMESCO Classification of Surgical Procedures) [24] procedure code for rectal resection, and diagnosis code C20 for rectal cancer according to the International Classification of Diseases version 10 (ICD-10) [25]. Due to some delay in data registration, and also to achieve at least 6 months follow-up, latest operation date was set to December 31, 2018. Tumors other than adenocarcinomas as well as transanal total mesorectal excision (TaTME) procedures were excluded. Data from NORGAST were linked via the patient’s individual social security numbers to the Norwegian Colorectal Cancer Registry [26] for information on preoperative work-up, oncological treatment upfront surgery, and final histopathological results.

### Statistical analyses

Data were analyzed with SPSS version 26 (IBM, Armonk, New York, USA). Differences between groups were assessed with Pearson’s chi square test for categorical data and two-sided *T*-test or Mann Whitney *U* test for continuous data. Confidence interval (CI), standard deviations, or quartiles were calculated as appropriate.

Survival and local recurrence were illustrated by Kaplan–Meier curves, and the log-rank test was used to test for difference between groups using an intention-to-treat factor approach. Adjusted survival and recurrence rates were further calculated using multivariable Cox regression analyses adjusting for baseline characteristics: gender, age, BMI, ECOG status as a measure of comorbidity and functional status, neoadjuvant chemoradiotherapy, operative technique, and clinical cancer stage. Information in the registries on possible comorbidity and functional status was available on ASA scores, preoperative pulmonary disease, cardiac disease, diabetes, and ECOG scores. A high degree of correlation was seen between these variables. ECOG scores were the only variable that significantly differed between ORR and LRR and was chosen for analyses.

To analyze the outcomes major complications, reoperations, and mortality within 30 postoperative days, a multivariable logistic model was built including the same variables for adjustment as mentioned above. Length of stay was analyzed with multivariable linear regression model, adjusting for the same variables as mentioned above.

There were some missing data in variables included for analyses. Little’s test [27]of whether data were missing completely at random was performed. The test had a non-significant *p*-value of 0.167 indicating that missing values were missing completely at random. This allowed patients with missing data in variables included for subgroup analyses to be excluded from these analyses, and complete case analyses were performed. To explore missing values even further, multiple imputations were done. A total of 5 imputations were created, and means of the 5 imputations were pooled into a new dataset via OMS (output management system). Survival analyses and regression analyses were rerun in the imputed dataset, and the results were essentially the same as with the complete case analyses.

### Variable definitions

Age was categorized into three groups (< 65 years, 65–80 years, and > 80 years). ASA scores were grouped into low (scores 1–2) and high (scores 3–4). ECOG scores were dichotomized into low (scores 0–1) and high (scores 2–4). Severe pulmonary disease was defined as having FEV1 < 50% or a vital capacity < 60% of predicted values. Severe cardiac disease was defined as NYHA classification 3–4 or severe arrhythmia requiring mechanical support. Complications were registered according to the Accordion grading system [28], and major complications were defined as Accordion grade 3 or higher. Anastomotic leak was defined as a leak requiring relaparoscopy/relaparotomy (grade C leak) [29]. Weight was classified by body mass index (BMI), and patients were grouped into 4 BMI classes [30]: [< 18.5], [18.5–25], [25–30], and [> 30]. Tumor level was measured in centimeters from anal verge with rigid proctoscope and categorized into three groups: low (0–5 cm), mid (5–10 cm), and high (10–15 cm) tumors.

## Results

**Fig. 1:**
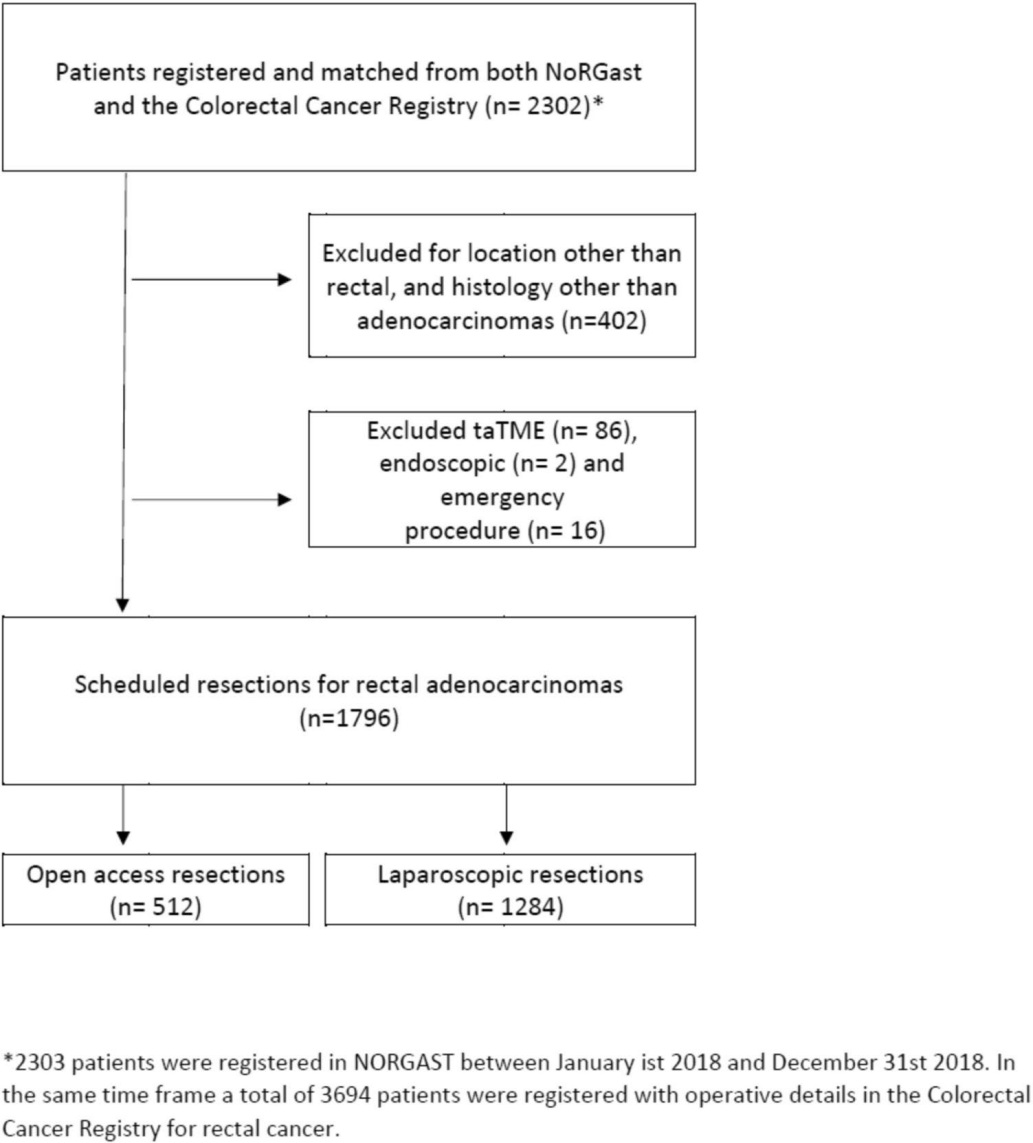
Patients who had undergone LRR including robotic-assisted procedures and ORR

From January 1, 2014, to December 31, 2018, a total of 2302 patients were recorded in NORGAST with rectal cancer and NCSP procedural code for rectal resection. During the same time frame, a total of 3694 patients were recorded in the Colorectal Cancer Registry^22^ with a major resection for rectal cancer, giving an overall coverage rate in NORGAST of 62%. After excluding patients with tumors other than adenocarcinoma, TaTME endoscopic and emergency procedures, a total of 1796 patients were included in this study. A total of 1284 patients had undergone LRR including 375 robotic-assisted procedures, and 512 had undergone ORR (Fig. 1). Conversion rate following laparoscopic procedures was 95/1284, 7.4%. A steady increase in laparoscopic procedures was observed during the study time frame, from 56% of the procedures registered in 2014 to 86% of the procedures in 2018. There were some baseline differences between the groups; patients who underwent ORR had higher ECOG scores and higher rates of severe pulmonary and cardiac disease (Table 1).

**Table 1:** Baseline characteristics

Baseline characteristics		Total	Laparoscopic	Open access	*p*-value
		*n* = 1796	*n* = 1284 (percentage)	*n* = 512	
Gender					
	Male	1108	782 (61.0)	326 (64.0)	0.276
	Female	688	502 (39.0)	186 (36.0)	
Age (avg) (std.dev)		67.3 (11.7)	67.5 (11.4)	66.6 (12.6)	0.997
BMI					
	< 18.5	40	25 (2.0)	15 (3.1)	0.354
	18–25	730	518 (41.1)	212 (43.6)	
	25–30	678	496 (39.4)	182 (37.4)	
	> 30	297	220 (17.5)	77 (15.8)	
Pulmonary disease		83	48 (3.7)	35 (6.8)	0.005
Heart disease		119	73 (5.7)	46 (9.0)	0.011
Diabetes		182	134 (10.4)	48 (9.4)	0.501
ASA score					
	Low (1–2)	1204	871 (67.8)	413 (69.9)	0.278
	High (3–4)	591	413 (32.2)	178 (34.8)	
ECOG score					
	Low (0–1)	1667	1210 (72.6)	457 (59.3)	0.002
	High (2–4)	111	67 (27.4)	46 (40.7)	
Radio(chemo)therapy		588	375 (29.2)	213 (41.6)	<0.001
Operative technique					
	LAR	1017	742 (57.8)	275 (53.7)	0.005
	APR	599	432 (33.6)	167 (32.6)	
	Hartmann	180	110 (8.6)	70 (13.7)	
cStage 1		303	246 (28.6)	57 (18.1)	<0.001
2		323	239 (27.8)	84 (26.7)	
3		399	288 (33.5)	111 (35.2)	
4		149	86 (10.0)	63 (20.0)	
cTumor x		156	82 (12.5)	74 (22.8)	< 0.001
1		74	60 (5.9)	14 (4.3)	
2		304	247 (24.3)	57 (17.5)	
3		636	473 (50.8)	163 (50.2)	
4		83	66 (6.6)	17 (5.2)	

There are missing values in some of the variables, listed under

BMI: 51 (laparoscopic; 25 open; 26)

ASA score: 1 (laparoscopic; 1 open; 0)

ECOG score: 16 (laparoscopic; 7 open; 9)

cStage: 622 (laparoscopic; 425 open; 197)

cTumor: 218 (laparoscopic; 150 open; 68)

### Long-term survival

**Fig. 2:**
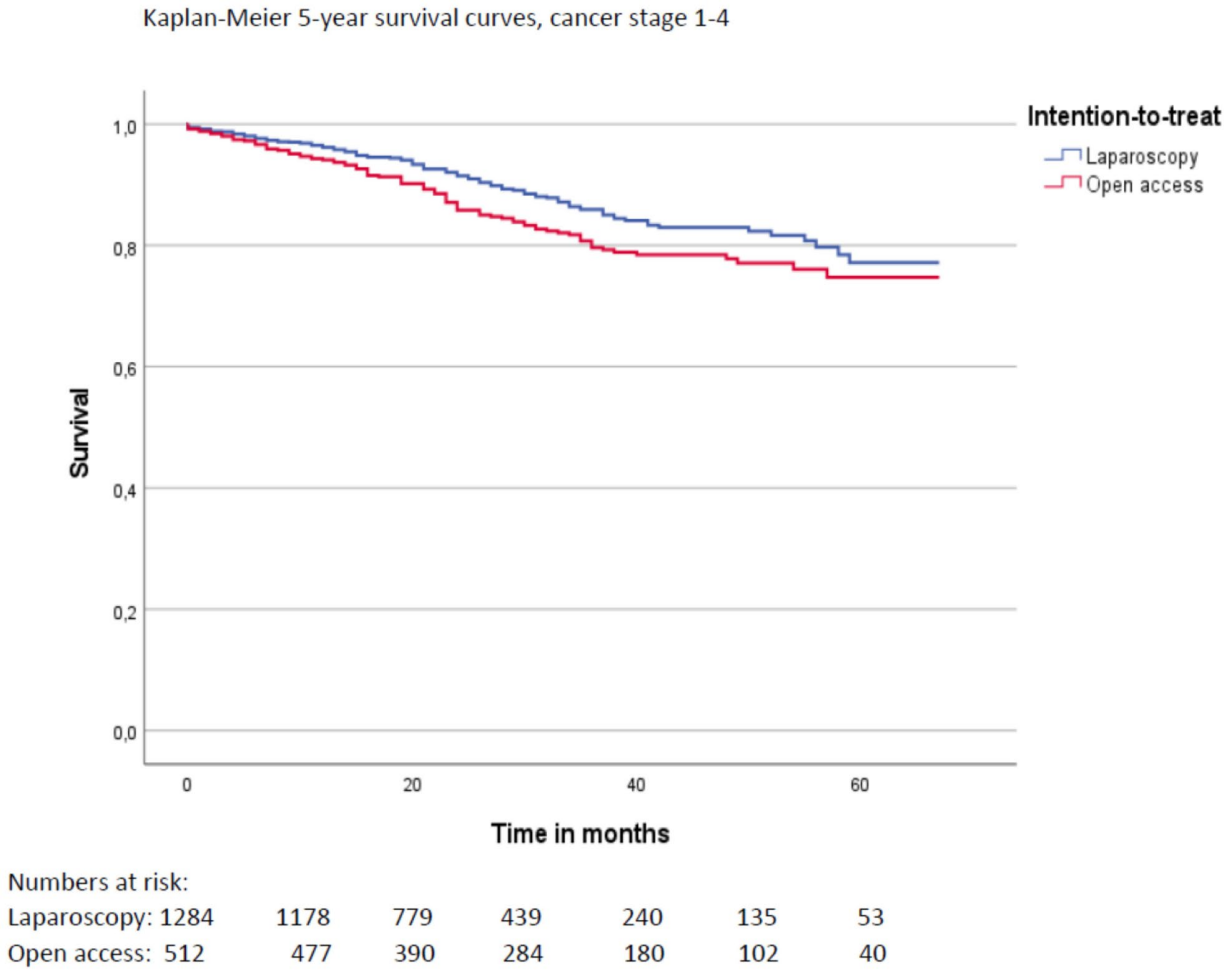
Overall 5-year survival for cancer stage 1–4 after LRR and after ORR

**Fig. 3:**
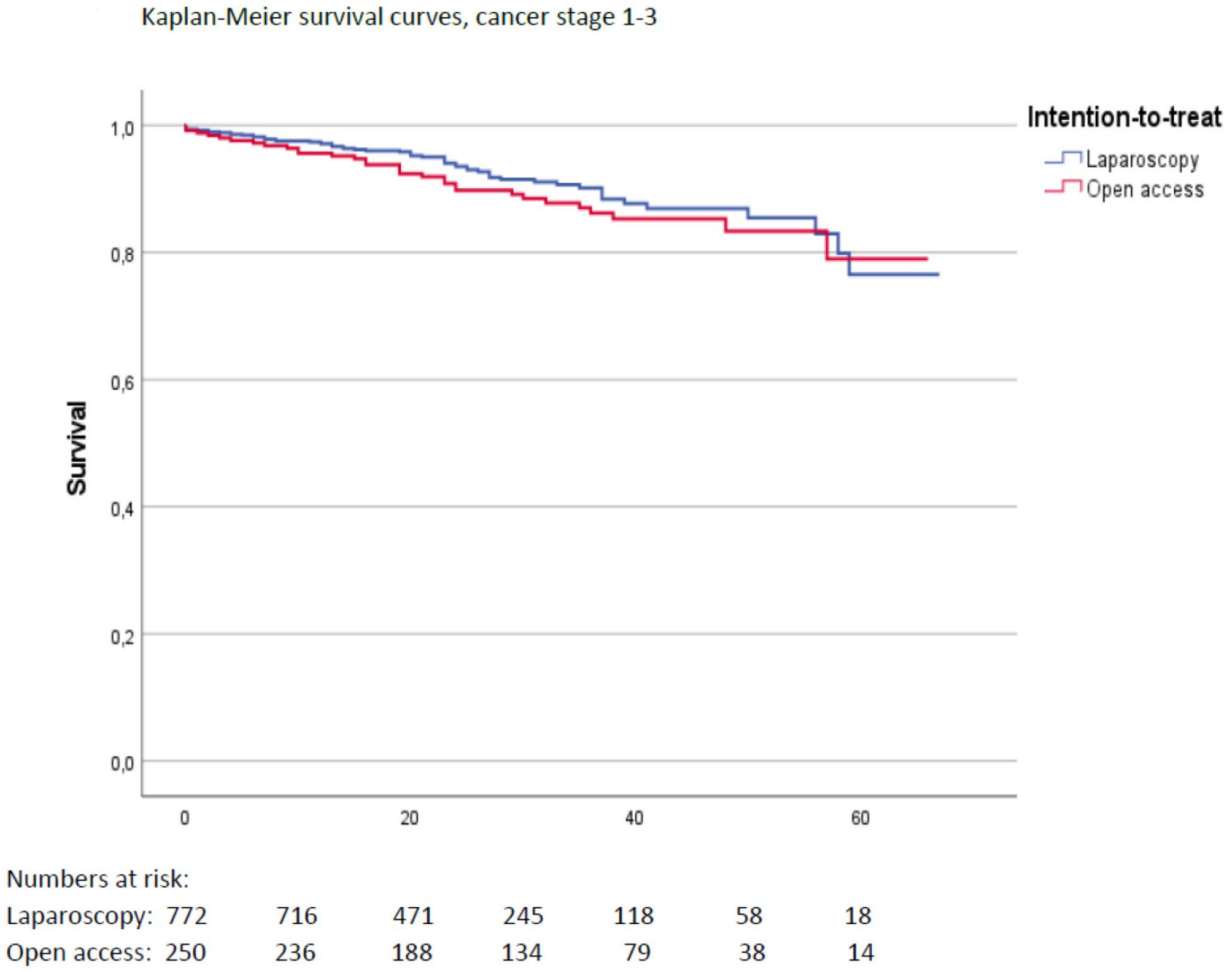
The 5-year survival for cancer stage 1–3 following LRR

Multivariable Cox regression analyses adjusting for clinical cancer stage, gender, age, ECOG score, BMI, access (ORR/LRR), operative procedure (APR, LAR or Hartmann procedure), tumor level, and preoperative radiochemotherapy as covariates showed no significant difference in HR between LRR and ORR (*p* = 0.386). Cancer stage 4 (aHR 4.19, 95% CI 2.17–8.12, *p* < 0.001) as well as increasing age (age > 80 years compared to < 65 years aHR 5.52, 95% CI 3.14–9.71, *p* < 0.001) and ECOG scores 3–4 compared to 1–2 (aHR 2.92, 95% CI 1.67–5.13, *p* < 0.001) was associated with increased long-term mortality hazard. Unadjusted overall 5-year survival for cancer stage 1–4 was 77.1% after LRR compared to 74.8% after ORR (*p* = 0.015, log rank test) (Fig. 2). For cancer stage 1–3, the 5-year survival was 76.5% following LRR compared to 79.0% following ORR (*p* = 0.670, log rank test) (Fig. 3). Missing values were missing completely at random according to Little’s test. After multiple imputations, the results were also essentially the same as for the complete case analyses.

### Local recurrence rates

**Fig. 4:**
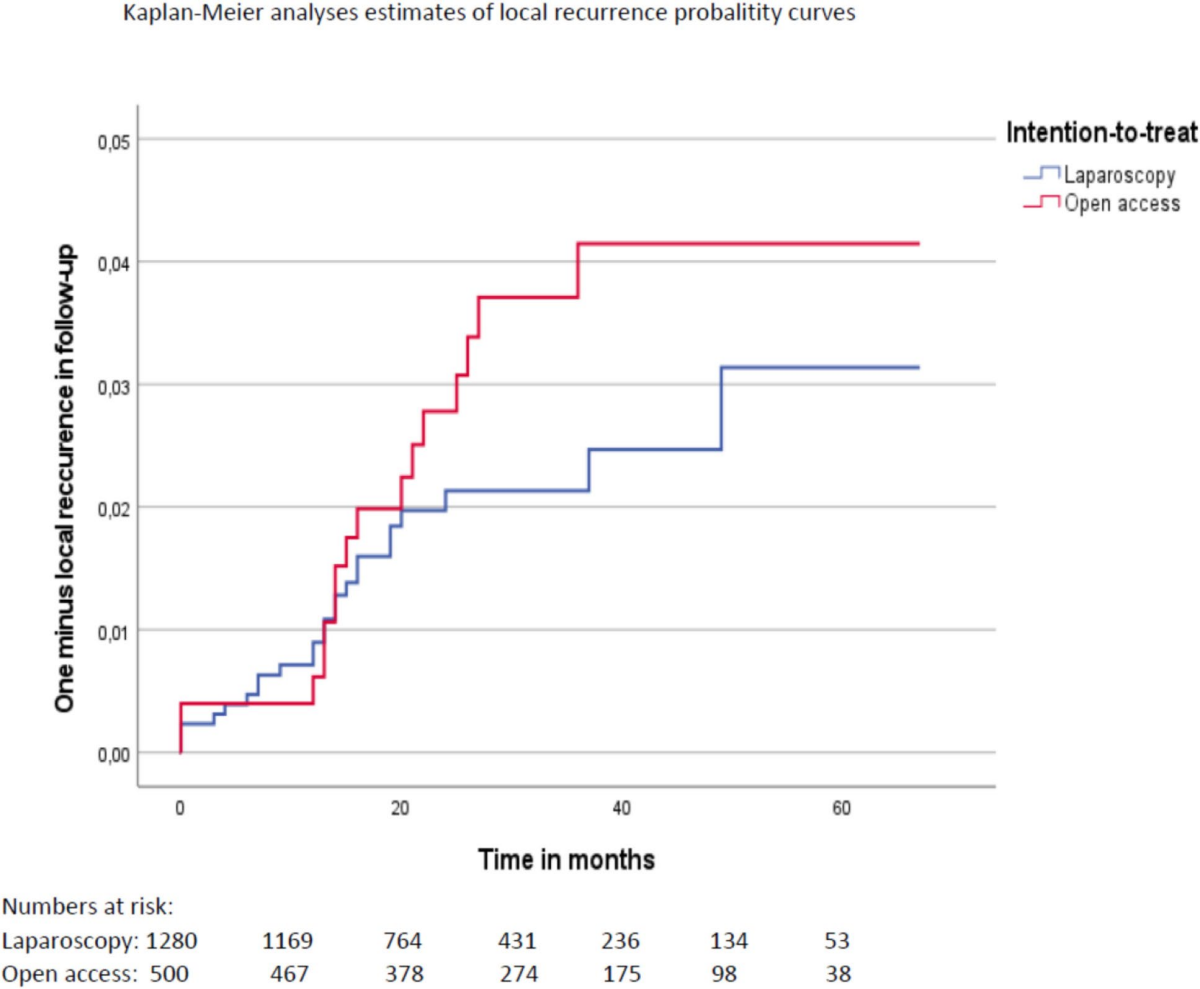
The 5-year rates of local recurrence following LRR and ORR

The 5-year rates of local recurrence were 3.1% following LRR and 4.1% following ORR (*p* = 0.249, log rank test) (Fig. 4). Multivariable Cox regression analyses including clinical cancer stage, age, ECOG score, BMI, access (ORR/LRR), operative procedure (APR, LAR, or Hartmann procedure), tumor level, and preoperative radiochemotherapy as covariates revealed no significant difference between the two groups for any covariates.

### Short-term outcomes

Length of hospital stay was median 6.0 (quartiles 4.0–8.0) days following LRR compared to 8.0 (quartiles 7.0–13.0) days following ORR (*p* < 0.001). Length of stay was further analyzed with multivariable linear regression, with age, gender, BMI, ECOG score, cancer stage, tumor level, preoperative radiochemotherapy, operative procedure, and access (ORR/LRR) as covariates. Access (ORR/LRR) was a significant predictor for the outcome length of stay, with an estimated additional length of stay for the ORR group of 3.1 days (*p* < 0.001, 95% CI 2.3–3.9). There were no other significant differences in short-term outcomes between the groups (Table 2).

**Table 2:** Short-term outcomes

**Complications within 30 days**			*All*	Laparoscopic, *n* = 1284	Open, *n* = 512	*p*-value
30-day mortality			*10*	5 (0.4)	5 (1.0)	0.131
Acc ≥ 3			*237*	155 (12.1)	82 (16.0)	0.026
Anastomotic leak*			*48 (1019)*	32 (742) (4.3)	16 (277) (5.8)	0.327
Reoperation			*144*	97 (7.6)	47 (9.2)	0.252
Length of stay	Median (quartiles)			6.0 (4.0–8.0)	8.0 (7.0–13.0)	<0.001
Single organ failure			*44*	27 (2.1)	17 (3.3)	0.132
Multi organ failure			*9*	5 (0.4)	4 (0.8)	0.288
**Histopathological results**						
CRM + **			*83*	51 (4.7)	32 (6.9)	0.079
DRM + ***			*9*	7 (0.7)	2 (0.4)	0.619
N. lymph nodes		Median (quartiles)		15.0 (12.0–20.0)	14.0 (12.0–19.0)	0.164

^*^Anastomotic leak rate calculated only on patients receiving an anastomosis. Number of patients receiving anastomosis in parenthesis

^**^Circumferential resection margin. Missing values in this variable: 240 (laparoscopic; 194 open; 46)

^***^Distal resection margin. Missing values in this variable: 265 (laparoscopic; 209 open; 56)

Numbers in parenthesis: percentages unless specified otherwise

Multivariable regression analyses did not show any difference in risk of major complications, reoperations or 30-day mortality between LRR and ORR (Table 2).

### Histopathological results

There were no differences between the access groups in rates of positive circumferential or distal resection margin nor number of harvested lymph nodes (Table 2).

## Discussion

The present study is based on compound data from two national quality registries covering the surgical and oncological quality of rectal cancer treatment in an unselected patient population and reflects national daily practice and true long-term results following rectal resection outside the strict frame of an RCT. The adjusted 5-year survival rates as well as 5-year local recurrence rates did not differ between the two groups. The length of stay differed significantly with an estimated LOS of 3 days longer after ORR compared to LRR.

Rectal cancer surgery has undergone significant changes during the last decades from the introduction of TME to minimally invasive surgery with laparoscopy, robotic-assisted surgery, and other approaches such as transanal total mesorectal excision. In part, this development has led to obvious advantages for the patients as complications such as surgical site infections [31], postoperative pain, development of incisional hernias, and scarring are more frequent following open than laparoscopic surgery [32–34]. However, despite widespread clinical implementation of laparoscopic access for rectal cancer surgery and the fact that multiple studies have been conducted to assess the results, a recent review [35] summarizing important studies concluded that the non-inferiority of laparoscopic as opposed to open resection in terms of pathological outcomes, local recurrence rates, and other long-term outcomes remains to be proven.

Only a few previous studies have explored long-term survival, oncological results, and complication rates following laparoscopic and open resection for rectal cancer. The CLASICC [13] trial was the first RCT comparing laparoscopic to open resection in 794 colorectal cancer patients, of whom more than half of the patients underwent surgery for rectal cancer. No difference in 5-year survival between open and laparoscopic rectal resections was found in intention-to-treat analysis, but patients who underwent conversion to open surgery had significantly reduced overall 5-year survival [13]. Patients who underwent anterior resection had higher rates of CRM positivity following LRR with 12% compared to 6% in the ORR group, although not statistically significant. Both 5-year local recurrence rate (10.1%) and distant recurrence rate (20.9%) did not differ between the groups. However, the conversion rate for rectal procedures was as high as 34%, and the CLASICC study has been criticized for being performed by many surgeons inexperienced with laparoscopic technique, as the only requirement was that participating surgeons should have had undertaken at least 20 laparoscopic colorectal resections prior to the study. This is supported by the steady decline in overall conversions from initially 38 to 16% at the end of the inclusion period [36], indicating that the results from the CLASICC study may be affected by surgeons’ learning curve in laparoscopic surgery.

The later COLORII study [37], a randomized controlled trial with 1044 included rectal cancer patients, showed comparable survival rates for LRR compared to ORR and with a local recurrence rate of 5.0% in both groups. The conversion rate in this study was 17%, but with no presented subgroup analysis on outcome after conversion. Nevertheless, intention-to-treat analysis revealed no difference in complication rates, completeness of mesorectum, number of harvested lymph nodes, or CRM positivity between the groups [37]. Also, in the COREAN [16] trial which included 340 patients who had undergone neoadjuvant chemoradiation therapy, no difference in CRM positivity or completeness of mesorectum was found between LRR or ORR and with similar 3-year survival. The 10-year results have recently been published, still with no difference in neither disease-free nor overall survival, and the authors concluded that laparoscopic procedure was non-inferior to open procedure.

In contrast the ALaCaRT study [12], a randomized multicenter study including 575 patients with rectal cancer T1-T3 failed to establish non-inferiority for LRR regarding completeness of mesorectum, CRM, and distal resection margin, although there were no significant differences between the open and laparoscopic group. At a median follow-up of 2 years, there were no difference in disease-free survival or local recurrence between LRR and ORR [38]. Similar results were found in the American ACOSOG-study [11, 39], which also concluded that non-inferiority for LRR could still not be established.

None the less a recent meta-analysis [40] of 12 randomized controlled trials comparing LRR and ORR in 3709 patients showed similar 5-year disease-free survival but significantly better overall survival after LRR.

The conversion rate of LRR has been a concern, as the CLASICC study showed inferior results in terms of increased complication rates and even worsened survival rates [13, 36]. Accordingly, previously published data from the present study cohort also identified an association between conversion and increased postoperative complication rate^41^. While the conversion rates in some older studies were above 15% [36, 37, 42], more recent studies report conversion rates between 1 and 12% [15, 41, 43, 44]. The introduction of robotic-assisted laparoscopy seems to further reduce the conversion rate in LRR. As intention-to-treat analyses have failed to show any inferior results following LRR as opposed to ORR, the risk of conversion cannot be used as an argument against laparoscopic access for rectal cancer surgery. The relatively low conversion rate in the present study, which was performed years after laparoscopy was introduced nationally for rectal cancer, indicates that the current results describe more matured laparoscopic surgery less affected by a learning effect.

This study has some limitations. As with all observational studies, variables that were not recorder may have potential confounding effects. Some baseline differences were observed between the groups, and Cox regression analyses were used to adjust survival rates for important differences. The variable clinical cancer stage had some missing values (622 out of 1796). Statistical tests show that missing data was missing completely at random meaning this variable is fit to include for further analyses, but results from analyses with this variable should be interpreted with caution. There was no information available in the registries on previous abdominal surgery or other reasons for expected adhesions/distorted anatomy that could demand open surgery. Unfortunately, there was no available information regarding type of neoadjuvant or adjuvant treatments given. Although long-course radiochemotherapy was the standard neoadjuvant regimen during the study period, some patients received short-course radiation alone or followed by chemotherapy. Furthermore, no information was available on whether “beyond TME-resections” or multivisceral resections had been performed, or whether the resections were performed with curative or palliative intention. Although only few patients undergo beyond TME resections or multivisceral resections for rectal cancer, this may still introduce a bias. In the present study, however, T4 tumors were operated more often by laparoscopic access than by open access (Table 1), which could have, if any, a negative impact on long-term survival in the laparoscopic access group rather than the open access group. Another limitation is that completeness of mesorectum was not available as a variable from the Norwegian Colorectal Cancer Registry. This is an important oncological quality measure of the surgical procedure along with circumferential and distal resection margins and number of lymph nodes harvested.

During the study period, total coverage in NORGAST compared to the Colorectal Cancer Registry was above 60%, which is acceptable. As a newly established register, the national coverage rates in NORGAST were low during the first years of the study period due to few participating hospitals. However, in-hospital coverage was high, with low risk for in-hospital selection bias.

The present study is one of the few studies that assesses several of the important aspects following LRR and ORR: long-term survival rate, long-term local recurrence rate, short-term complication rate including hospital length of stay, reoperations, anastomotic leak rates, and histopathological results. Results after LRR were similar compared to ORR, but with significantly shorter hospital length of stay. Thus, the present study supports the view that laparoscopy should be chosen over open access for rectal cancer resection if no specific reason to choose otherwise exists, such as known adhesions, severe pulmonary disease, or other challenges, such as locally advanced tumors affecting adjacent tissues.

### Supplementary Information

Below is the link to the electronic supplementary material.

Supplementary file1 (DOCX 27 KB)

## Data Availability

The data that supports the findings of this study are not publicly available due to privacy restrictions of the research participants.
