# Supplementary material for: Laparoscopic rectal cancer resection yields comparable clinical and oncological results with shorter hospital stay compared to open access: a 5-year national cohort
Source: Int J Colorectal Dis. 2023 Oct 4;38(1):247. doi: 10.1007/s00384-023-04529-z (PMC10550871; doi:10.1007/s00384-023-04529-z)
Supplement: Supplementary file 1 — Supplementary file1 (DOCX 27 KB) [file 384_2023_4529_MOESM1_ESM.docx]

Results from multivariable logistic regression analyses

| **Outcome measure** | **Significant variables** | | | | |  | **Univariable analyses** | | | **Multivariable analyses** | |
| --- | --- | --- | --- | --- | --- | --- | --- | --- | --- | --- | --- |
|  | **Variable** | | | |  | **Rate** | **OR (95% c.i.)** | | **p-value** | **aOR (95% c.i.)** | **p-value** |
| **Major complications** | Age group | | | | <65 | *14,2 %* | Ref | | 0.227 |  |  |
|  |  | | | | 65-80 | *12,5 %* | 0.86 (0.65-1.15) | |  |  |  |
|  |  | | | | >80 | *10,3 %* | 0.67 (0.41-1.08) | |  | Ref | 0.005 |
|  | Gender | | | | Male | *15,3 %* | 1.85 (1.36-2.51) | |  | 1.78 (1.19-2.66 | |
|  |  | | | | Female | *9,1 %* | Ref | | <0,001 |  |  |
|  | ECOG-score | | | | 1-2 | *12,9 %* | Ref | | 0.770 |  |  |
|  |  | | | | 3-4 | *14,4 %* | 1.09 (0.63-1.88) | |  |  |  |
|  | BMI-group | | | | <18,5 | *10,0 %* | 1.00 (0.35-2.89) | | 0.003 | 0 | 0.008 |
|  |  | | | | 18,5-25 | *9,8 %* | Ref | |  | Ref |  |
|  |  | | | | 25-30 | *13,8 %* | 1.49 (1.07-2.05) | |  | 1.11 (0.74-1.68) | |
|  |  | | | | >30 | *18,0 %* | 2.00 (1.37-2.93) | |  | 2.16 (1.35-3.44) | |
|  | Operative tech. | | | | LAR | *14,2 %* | Ref | | 0.157 |  | 0.216 |
|  |  | | | | Hartmann | *13.7%* | 1.02 (0.65-1.59) | |  | 0.49 (0.23-1.08) | |
|  |  | | | | APR | *10.5%* | 0.75 (0.55-1.02) | |  | 0.92 (0.63-1.35) | |
|  | Tumor level | | | | Low | *9,9 %* | Ref | | 0.243 |  |  |
|  |  | | | | Mid | *13,2 %* | 1.44 (0.91-2.26) | |  |  |  |
|  |  | | | | High | *10,8 %* | 1.13 (0.69-1.84) | |  |  |  |
|  | Radiochemo | | | | Yes | *11,5 %* | 0.86 (0.64-1.16) | | 0.328 |  |  |
|  |  | | | | No | *13,6 %* | Ref | |  |  |  |
|  | Cancer stadium | | | | 1 | *10.6%* | Ref | | 0.044 | Ref | 0.013 |
|  |  | | | | 2 | *14.2%* | 1.41 (0.87-2.28) | |  | 1.39 (0.84-2.29) | |
|  |  | | | | 3 | *10.8%* | 1.02 (0.63-1.66) | |  | 1.03 (0.63-1.69) | |
|  |  | | | | 4 | *18.8%* | 1.96 (1.13-3.39) | |  | 2.31 (1.30-4.12) | |
|  | Access | | | | Laparoscopy | *12,1 %* | Ref | | 0.026 |  | 0.365 |
|  |  | | | | Open | *15,2 %* | 1.39 (1.04-1.86) | |  | 1.20 (0.81-1.78) | |
|  | | | **Variable** |  | | ***Rate*** | **OR (95% c.i.)** | **p-value** | | **aOR (95% c.i.)** | **p-value** |
| **30-day  mortality** | | | Age group | <65 | | *0,1 %* | Ref | 0.008 | | Ref | 0.028 |
|  | | |  | 65-80 | | *0,4 %* | 3.31 (0.37-29.67) |  | | 2.56 (0.26-25.27) | |
|  | | |  | >80 | | *2,3 %* | 16.89 (1.96-145.41) | | | 12.88 (1.36-121.65) | |
|  | | | Gender | Male | | *0.5%* | 1.45 (0.37-5.63) |  | |  |  |
|  | | |  | Female | | *0.4%* | Ref | 0.590 | |  |  |
|  | | | ECOG-score | 1-2 | | *0,3 %* | Ref | <0,001 | | Ref | 0.012 |
|  | | |  | 3-4 | | *3,6 %* | 10.16 (2.83-36.54) |  | | 6.58 (1.50-28.78) | |
|  | | | BMI-group | <18,5 | | *5,0 %* | 19.16 (2.63-139.72) | 0.011 | | 9.01 (1.09-74.07) | 0.073 |
|  | | |  | 18,5-25 | | *0,3 %* | Ref |  | | Ref |  |
|  | | |  | 25-30 | | *0,3 %* | 1.08 (0.15-7.67) |  | | 1.56 (0.21-11.44) | |
|  | | |  | >30 | | *0,7 %* | 3.71 (0.62-22.34) |  | | 6.76 (1.04-43.78) | |
|  | | | Operative tech. | LAR | | *0,5 %* | Ref | 0.973 | |  |  |
|  | | |  | Hartmann | | *0,6 %* | 0.94 (0.11-7.87) |  | |  |  |
|  | | |  | APR | | *0,5 %* | 0.85 (0.21-3.40) |  | |  |  |
|  | | | Tumor level | Low | | *0,0 %* | 0 | 0.590 | |  |  |
|  | | |  | Mid | | *0,8 %* | N.A. |  | |  |  |
|  | | |  | High | | *0,3 %* | N.A. |  | |  |  |
|  | | | Radiochemo | Yes | | *0,3 %* | 0.88 (0.23-3.42) | 0.853 | |  |  |
|  | | |  | No | | *0,6 %* | Ref |  | |  |  |
|  | | | Cancer stadium | 1 | | *0.7%* | Ref | 0,943 | |  |  |
|  | | |  | 2 | | *0.3%* | 0.47 (0.04-5.18) |  | |  |  |
|  | | |  | 3 | | *0.5%* | 0.76 (0.11-5.42) |  | |  |  |
|  | | |  | 4 | | *0 %* | 0 |  | |  |  |
|  | | | Access | Laparoscopy | | *0,4 %* | Ref | 0.145 | |  | 0.412 |
|  | | |  | Open | | *0,8 %* | 2.52 (0.72-8.75) |  | | 1.78 (0.45-7.05) | |
|  | | **Variable** | | |  | ***Rate*** | **OR (95% c.i.)** | **p-value** | | **aOR (95% c.i.)** | **p-value** |
| **Reoperations** | | Age group | | | <65 | *8,6 %* | Ref | 0.234 | |  |  |
|  | |  | | | 65-80 | *8,1 %* | 0.94 (0.66-1.34) |  | |  |  |
|  | |  | | | >80 | *5,2 %* | 0.56 (0.29-1.09) |  | |  |  |
|  | | Gender | | | Male | *9,6 %* | 1.95 (1.32-2.88) |  | | 1.99 (1.33-2.98) | |
|  | |  | | | Female | *5,3 %* | Ref | <0.001 | | Ref | 0.001 |
|  | | ECOG-score | | | 1-2 | *7,9 %* | Ref | 0.742 | |  |  |
|  | |  | | | 3-4 | *9,0 %* | 1.12 (0.57-2.19) |  | |  |  |
|  | | BMI-group | | | <18,5 | *5,0 %* | 0.77 (0.17-3.27) | 0.151 | | 0.95 (0.22-4.14) | 0.373 |
|  | |  | | | 18,5-25 | *6,5 %* | Ref |  | | Ref |  |
|  | |  | | | 25-30 | *8,7 %* | 1.41 (0.95-2.09) |  | | 1.23 (0.82-1.84) | |
|  | |  | | | >30 | *9,9 %* | 1.63 (1.01-2.64) |  | | 1.53 (0.94-2.49) | |
|  | | Operative tech. | | | LAR | *10,0 %* | Ref | <0,001 | | Ref | 0.009 |
|  | |  | | | Hartmann | *7,4 %* | 0.69 (3.83-1.27) |  | | 0.76 (0.42-1.39) | |
|  | |  | | | APR | *4,6 %* | 0.46 (0.29-0.69) |  | | 0.49 (0.32-0.78) | |
|  | | Tumor level | | | Low | *4,8 %* | Ref | 0.218 | |  |  |
|  | |  | | | Mid | *7,8 %* | 1.69 (0.91-3.13 |  | |  |  |
|  | |  | | | High | *7,7 %* | 1.64 (0.86-3.11) |  | |  |  |
|  | | Radiochemo | | | Yes | *5,2 %* | 0.54 (0.36-0.81) | 0.003 | |  | 0.078 |
|  | |  | | | No | *9,3 %* | Ref |  | | 0.68 (0.44-1.04) | |
|  | | Cancer stadium | | | 1 | *6.3%* | Ref | 0.668 | |  |  |
|  | |  | | | 2 | *7.1%* | 1.15 (0.61-2.15) |  | |  |  |
|  | |  | | | 3 | *6.8%* | 1.08 (0.59-1.99) |  | |  |  |
|  | |  | | | 4 | *9.4%* | 1.55 (0.75-3.19) |  | |  |  |
|  | | Access | | | Laparoscopy | *7,5 %* | Ref | 0.253 | |  |  |
|  | |  | | | Open | *9,0 %* | 1.24 (0.86-1.78) |  | |  |  |
